# Machine-Learning-Based Atomistic Model Analysis on High-Temperature Compressive Creep Properties of Amorphous Silicon Carbide

**DOI:** 10.3390/ma14071597

**Published:** 2021-03-25

**Authors:** Atsushi Kubo, Yoshitaka Umeno

**Affiliations:** Institute of Industrial Science, The University of Tokyo, Tokyo 113-8654, Japan; umeno@iis.u-tokyo.ac.jp

**Keywords:** silicon carbide, ceramic matrix composites, creep properties, high-temperature strength, molecular dynamics, artificial neural network

## Abstract

Ceramic matrix composites (CMCs) based on silicon carbide (SiC) are used for high-temperature applications such as the hot section in turbines. For such applications, the mechanical properties at a high temperature are essential for lifetime prediction and reliability design of SiC-based CMC components. We developed an interatomic potential function based on the artificial neural network (ANN) model for silicon-carbon systems aiming at investigation of high-temperature mechanical properties of SiC materials. We confirmed that the developed ANN potential function reproduces typical material properties of the single crystals of SiC, Si, and C consistent with first-principles calculations. We also validated applicability of the developed ANN potential to a simulation of an amorphous SiC through the analysis of the radial distribution function. The developed ANN potential was applied to a series of creep test for an amorphous SiC model, focusing on the amorphous phase, which is expected to be formed in the SiC-based composites. As a result, we observed two types of creep behavior due to different atomistic mechanisms depending on the strain rate. The evaluated activation energies are lower than the experimental values in literature. This result indicates that an amorphous region can play an important role in the creep process in SiC composites.

## 1. Introduction

From the viewpoint of structural materials, silicon carbide (SiC) is used as the matrices and reinforcing fibers in the ceramics matrix composites (CMCs). The CMC whose matrix and fiber consist of SiC is called SiC/SiC. The SiC/SiC composite has prominent mechanical properties such as light weight, high elastic modulus, high strength, and high fracture toughness [1,2], and these features makes the SiC/SiC composites applicable to the hot section in turbines [3,4]. For such application of SiC-based structural materials, the mechanical properties at high temperature are of crucial importance and have been investigated by experiments (e.g., the temperature dependence of tensile strength and creep properties for a SiC fiber [5,6], and high-temperature fatigue behavior of a SiC/SiC composite [7]). Since such experiments require a lot of time and special experimental equipment, assistance by numerical simulation approaches is necessary for evaluation and analysis of the mechanical properties. The molecular dynamics (MD) simulation can be a powerful tool to investigate the essential mechanisms of deformation and fracture, if a suitable interatomic potential function is available. Moreover, ideally, the material properties obtained by the MD simulations can be used as the material parameters for larger-scale simulations; e.g., the activation energies of creep and diffusion are applied to the phase field simulations, the finite element method (FEM) analysis, etc.

The reliability of MD simulation is mainly dependent on the quality of the applied potential function, and thus the potential function should be chosen (or developed) properly according to the purpose of the simulation. Thus far many potential functions have been developed for the Si-C system based on various physical models such as the bond-order model [8,9], the modified embedded atom model [10], the ionic model [11,12], and the charge-transfer model [13]. Nevertheless, there are still some non-trivial difficulties in development of an interatomic potential function for the high-temperature analysis of SiC, as follows:

1. In SiC fibers and matrices, the ratio of Si and C atoms is *not* necessarily stoichiometric but may be varied. For example, Hi-Nicalon, a typical SiC fiber, consists of Si and C of 39 at% and 60.4 at%, respectively [6]. Therefore, a constant-charge model, such as Vashishta et al. [11] and Kubo et al. [12], is not applicable any longer.
2. Unlike a low-temperature condition, the local atomic structure can easily change at a high temperature via, e.g., creep and diffusion processes. In such situation, the bond-order type potential functions may cause qualitative and quantitative errors, because in general such potential functions tend to overestimate the bonding energy or critical force of bond breaking.
3. It is also unclear what interactions are dominant on the mechanical properties of SiC at high temperature. In SiC, both ionic and covalent interactions are competitive and thus play an important role in deformation and fracture in SiC. That is true even in the case of a perfect crystal of SiC at 0 K, where cleavage, slip, and phase transition were observed with a slight difference in the loading condition in a first-principles (FP) analysis [14]. This complex feature of the interaction in SiC requires a highly flexible and versatile formulation in the potential model.
4. The practical SiC materials include other types of atoms (e.g., B, O, N, Al, etc.) as impurities and/or dopants [15,16] (Relatedly, ceramic fibers consisting of Si, B, N, and C also have been produced [17]). Therefore, the potential function for the Si-C systems should be extendable to a many-species system for further investigation. The existence of such impurities and/or dopants also requires a flexible formulation of the potential model because the additional atoms can be metallic, covalent, or ionic.

In short, MD simulations of the high-temperature mechanics of SiC require the interatomic potential model to be highly transferable, flexible and extendable. It is difficult to address all these problems by means of conventional interatomic potential models (Even if possible, it may be accompanied by a substantial modification of the function forms). In contrast, the interatomic potential models based on machine-learning approaches can be a suitable solution to overcome these problems because of their systematic formulations. Several types of machine-learning potentials have been thus far proposed [18], e.g., the artificial neural network (ANN) model [19,20], the Gaussian approximation potential [21], the gradient-domain machine learning [22], and other approaches [23]. Especially the artificial neural network (ANN) potential models have been widely applied because of its flexibility. In a mathematical sense, an ANN can mimic any function with a given accuracy [24,25,26]. In a physical sense, an ANN has no physical background and thus can be applied to any type of materials including metals [27,28], metal oxides [20,29], carbon polymorphs [30,31], and organic molecules and complexes [32]. In addition, application of the ANN models to atomistic analyses is not limited to use as a potential function. Very recently, several ANN applications have been reported, where ANNs are used for prediction of electronic properties in the atomic systems [33,34,35]. Thanks to a lack of the physical background in ANN models, a framework of the ANN potential function is applicable to other objects, e.g., a prediction of the electronic density of state, without any special modification in the ANN architecture itself, as was demonstrated by Umeno and Kubo [33].

In this study, we develop an ANN potential function for the Si-C systems, for the purpose of investigation of high-temperature mechanical properties in the SiC structural materials. While real SiC-based ceramics contain other atomic species, we omit such impurity or dopant atoms and focus on pure Si-C systems for simplicity. Some mechanical properties are evaluated for SiC single crystals for the purpose of validation of the ANN potential. After that, the ANN potential function is applied to high-temperature deformation analyses. On performing MD simulations, it is required to set a reasonable simulation model of atomic structure because a whole CMC system cannot be examined by molecular simulations owing to the limitation of the length scale. According to experimental observations, the grain boundary region plays an important role in the high-temperature mechanical behavior such as creep in ceramics [6,36]. Thus, we mainly examine the amorphous-phase structures to mimic the non-crystalline grain boundary regions instead of directly investigating a CMC structure or a polycrystal model for simplicity and efficiency of simulation; i.e., we regard the amorphous model as the limit of small-grain. The effect of oxygen atoms, which is a major cause of forming the amorphous phase, is also omitted for simplicity. The creep properties of amorphous SiC is evaluated as a relationship among the strain rate, stress, and temperature. The mechanisms of creep in the amorphous SiC are discussed by analyzing the structural change during deformation.

This study is organized as follows. In Section 2, we introduce the potential function based on the ANN model and explain the procedure of parametrization. In Section 3, the developed ANN potential is validated through several simple MD simulations. Typical material properties such as the lattice constant are compared with the FP calculations and experiments in literature. In Section 4, we apply the ANN potential to practical analyses to evaluate the high-temperature mechanical properties, especially the creep properties under a variety of loading conditions. Lastly, in Section 5, we summarize the results and make the concluding remarks.

## 2. Construction of Artificial Neural Network Potential Model

### 2.1. Formulation

**Figure 1:**
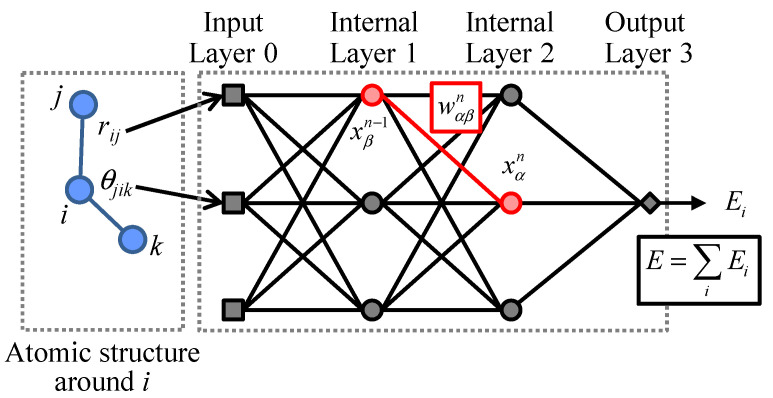
Schematic illustration of ANN potential model. This figure shows an exemplified network model with two internal layers (layers 1 and 2) and three nodes each in the input and internal layers.

We adopted the framework of the ANN potential model proposed by Behler and Parrinello [19], where the potential energy for each atom is evaluated as a function of the local atomic structure within the cutoff radius. The ANN architecture is schematically shown in Figure 1. An ANN model is composed by the input layer (layer 0), internal layers (layers 1, ..., *N* − 1) and the output layer (layer *N*). Each layer consists of nodes, whose state is expressed by a real number. The state of the nodes in the input layer is determined from local atomic structure by the basis functions. The output layer has a single node and, its state is returned as the potential energy. The state of the *α*-th node in the *n*-th layer, $x_{\alpha }^{n}$ (*n* > 0), is determined with the nodes in the previous layer *n*−1 as follows:

(1) $$x_{\alpha }^{n}=f_{a}^{n}\left(\sum_{\beta =1}^{M_{n-1}}w_{\alpha \beta }^{n}x_{\beta }^{n-1}+w_{\alpha 0}^{n}\right),$$

where $w_{\alpha \beta }^{n}$ denotes the weight parameter, and $w_{\alpha 0}^{n}$ is the bias weight parameter independent of the state of the previous layer. Those weight parameters are to be optimized through machine learning. The function $f_{a}^{n}$ is the activation function for the *n*-th layer. The controllable architectural parameters in this model are the number of the internal layers *N*, the number and types of the basis functions, and the number of nodes in each layer *M_n_* for *n* = 1, ..., *N* − 1 (the number of nodes in the input layer, *M*_0_, is equal to the number of the basis functions). In addition, also the type of the activation function $f_{a}^{n}$ (*n* = 1, ..., *N* − 1) can be changed.

While the original Behler-Parrinello model applies an exponential-type basis function set, we adopted the basis function set formulated by Artrith et al. [32] This basis set includes the two-body interaction *φ_μ_*(*r*) and the three-body interaction *ψ_μ_*(*θ*):

(2) $$\phi_{\mu }(r)=T_{\mu }\left(\frac{2r}{r_{c}}-1\right),$$

(3) $$\psi_{\mu }(\theta )=T_{\mu }\left(\cos\theta \right),$$

where the series of *T_μ_*(*x*) (*x*∈[−1, 1], *μ* = 0, 1, 2, ...) is the Chebyshev polynomials of the first kind defined recursively as

(4) $$\left\{\begin{array}{l} T_{0}(x)=1,\\ T_{1}(x)=x,\\ T_{\mu +1}(x)=2xT_{\mu }(x)-T_{\mu -1}(x). \end{array}\right.$$

Using these basis sets, the state of the nodes in the input layer, *x*^0^*_α_*, is determined as follows:

(5) $$x_{\alpha }^{0\;(\mathrm{pair})}=\sum_{j}\phi_{\alpha -1}(r_{ij})f_{c}(r_{ij}),$$

(6) $$x_{\alpha }^{0\;(\mathrm{angle})}=\sum_{j,k}\psi_{\alpha -1}(\theta_{jik})f_{c}(r_{ij})f_{c}(r_{ik}),$$

where *f*_c_ is the cutoff function to truncate the basis functions at the cutoff radius *r*_c_. In this formulation, each of two-body and three-body basis functions has only two empirical parameters, i.e., the number of terms for expansion, *N*_2_ and *N*_3_, and the cutoff radius *r*_c_. This simple feature enables a systematic expansion of the basis function.

The activation function $f_{a}^{n}$ is given as

(7) $$f_{a}^{n}(x)=\left\{\begin{array}{ll} 1.7159\tanh\left(\frac{2x}{3}\right)+0.1x & \left(n=1,\ldots ,N-1\right)\\ x & \left(n=N\right) \end{array}\right..$$

While the cutoff function *f*_c_ is given by a cosine function in the original model [32], we modified the cutoff function to the following form:

(8) $$f_{c}(r)=\left\{\begin{array}{cc} \frac{1}{2}\left[1+\sin\left(\frac{\pi }{2}\cos\left(\frac{\pi r}{r_{c}}\right)\right)\right] & \left(r\leq r_{c}\right)\\ 0 & \left(r>r_{c}\right) \end{array}\right..$$

The main purpose of this modification is to make the basis functions smoother at the cutoff radius *r*_c_.

An individual ANN model is assigned for each atomic species, i.e., Si and C, with the identical architecture. The architectural parameters of the basis functions are summarized in Table 1. The number of nodes in the input layer is equal to the number of terms of basis functions, i.e., 44 (16 terms for two pair types and 4 terms for three triplet types). Each ANN possesses two internal layers with 20 nodes each. Therefore, each ANN model has 1341 parameters ($w_{\alpha \beta }^{n}$ and $w_{\alpha 0}^{n}$) to be optimized.

### 2.2. Optimization Procedure and Reference Data

The internal parameters in the ANN potential were optimized in order that the ANN model can reproduce the relationship between the atomic structure and the potential energy in the target material system. We refer to this relationship or mapping as the reference data, and the atomic structures for the reference data are called the reference structures. We obtained the reference data by the FP calculation based on the density functional theory (DFT) [37], which can reproduce the basic material properties such as the lattice constants and elastic constants for the Si-C systems [12,14] and deal with relatively large number of atoms compared with other FP approaches.

The optimization was carried out in the following way: Firstly, the initial reference data set was obtained for basic atomic structures. We conducted DFT calculations for the stable atomic structures of SiC (2H, 3C), Si (diamond), and C (diamond, graphene). In addition, we collected the reference data for other typical structures of high symmetry (e.g., bcc, fcc, etc.) and the strained structures, where affine deformation is applied up to 20% in typical modes. The structures and deformation modes are shown in Appendix A.

Secondly, the ANN parameters were optimized to develop a provisional ANN potential function. Here, the architecture of the ANN model (the number of layers, the number of nodes, etc.) was not changed during optimization. We chose 90% of the reference data at random and used them as the training data. The remaining 10% was left as the test data for validation.

**Figure 2:**
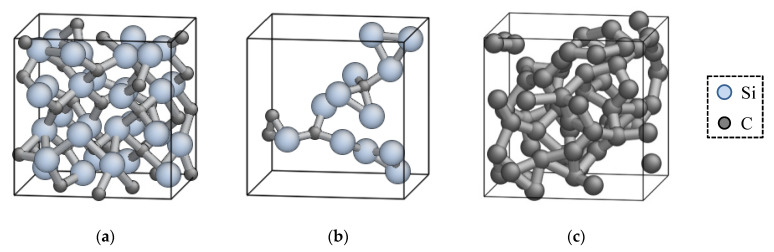
Example of reference structures obtained by MD simulation using provisional ANN potential functions. (**a**) 3C-SiC crystal near the melting point; (**b**) non-stoichiometric sparce Si-C system; (**c**) fused C system.

Thirdly, several MD simulations were performed using the provisional ANN potential to confirm its validity in qualitative and quantitative aspects. If the ANN potential can reproduce all the material properties of interest, the optimization process is over. However, an atomic structure often falls into an unrealistic structure during MD simulation, especially at an early stage of the optimization cycle. If impermissible errors or unphysical behavior (e.g., unrealistic phase transition) are found in the ANN results, the atomic structures with such a disagreement are added to the reference data set, and the ANN potential is optimized again with the additional reference data. This series of development cycle (addition of reference data, optimization, and validation) was repeated until the resultant ANN potential reaches an acceptable level of quality. This method can selectively detect atomic structures for which the ANN returns an erroneous result, and thus enables an efficient optimization of the potential function [12,38]. For this purpose, we also examined untypical atomic structures that are obtained from the MD simulations under ultimate conditions, such as clusters consisting of a small number of atoms, structures fused at an extremely high temperature, etc. These data can enhance the transferability of the resultant ANN potential. Exemplified atomic structures obtained via this process are shown in Figure 2.

Note that the potential energy tends to be underestimated in the atomic structures obtained by the MD simulation with a provisional ANN potential. That is simply because an atomic structure is unlikely to appear during MD simulation if its potential energy is overestimated. This trend inevitably causes a bias in the reference structures and indicates a limitation of the abovementioned approach (this problem is *not* peculiar to the ANN potential model or other machine-learning potentials but common to any type of the potential models). To address this problem, we also collected the reference structures in another way. We picked up several atomic structures resulting from the MD simulations, and those structures were relaxed by the DFT calculation. The fully relaxed structure and transient structures during relaxation were used as the reference structures, and the potential energy of those structures is likely to be overestimated by the provisionally developed ANN potential. Since this approach requires more computational cost for relaxation by DFT calculation, relatively fewer reference data were generated by this method.

All the DFT calculations were conducted by Vienna Ab-initio Simulation Package (VASP) [39,40] with the generalized gradient approximation (GGA) by Perdew et al. [41] The core electrons were dealt with by the projector augmented wave (PAW) method [42]. The ANN potential was developed by the Atomic Energy Network (ænet) package [20] with the limited-memory Broyden-Fletcher-Goldfarb-Shanno method [43]. The MD simulations were carried out by the LAMMPS code [44,45] on which the ænet library was implemented by Mori [46]. The numbers of the reference structures for training and testing were 18,571 and 2063 in total, respectively.

## 3. Validation of ANN Potential Function

### 3.1. Result of Optimization

**Figure 3:**
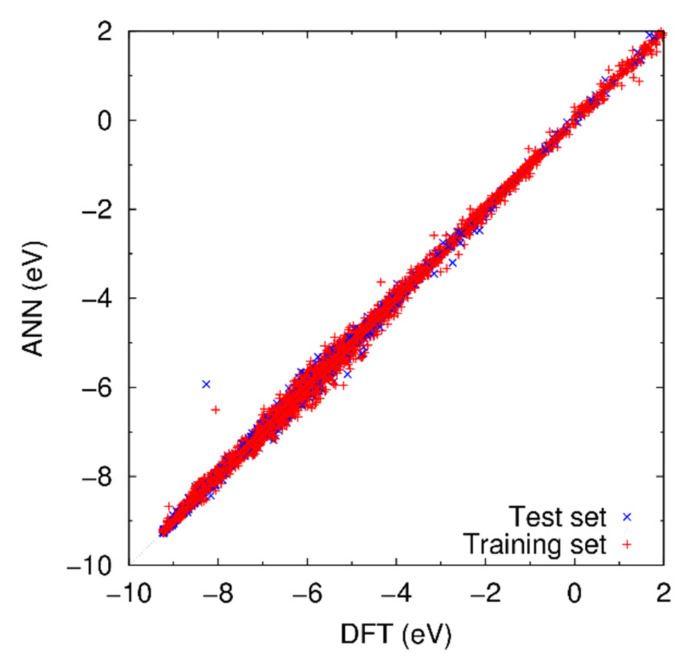
Potential energy of reference structures obtained by ANN potential and DFT calculation. Note that there are data points with the potential energy beyond +2.0 eV, which are not shown here because of less importance.

Figure 3 shows the comparison of the potential energies of the reference structures calculated by the DFT calculation and the developed ANN potential. Each data point corresponds to one reference structure. If the potential energy of a reference structure is consistent with the ANN and DFT calculations, the corresponding data point locates near the diagonal line, *y* = *x*. Almost all the data points in Figure 3 are found to locate near the diagonal line, and thus the developed ANN potential is expected to be able to reproduce the DFT results for various atomic structures. The ANN potential overestimates the potential energy of some reference structures, which are amorphous structures composed by carbon. Thus, the ANN potential may cause not a little error for the simulations with such structures. Although this issue should be addressed eventually, such error is still acceptable for the present purpose, because (i) the amorphous phases of pure carbon is out of our scope and not close to our target, and (ii) overestimation of the potential energy is much less likely to cause a fatal problem than underestimation, which can induce an unrealistic phase transition.

### 3.2. Material Properties at Equilibrium States

Table 2 lists the lattice constants, potential energies, and elastic constants at equilibrium state of the typical crystal structures of SiC, Si, and C obtained by the ANN potential and DFT calculation.

It is found that the ANN potential can evaluate the lattice constants and the potential energy of typical crystal structures in a good agreement with the DFT calculation. Remarkably, the ANN potential can successfully reproduce the small energy gaps between the 3C and 2H structures of SiC, and the diamond and graphene structures of C. This result indicates that the developed ANN potential is applicable to a variety of atomic environment. On the other hand, the elastic constants are found to have relatively large error from the DFT results. This is mainly because the elastic constants are related to the second derivative of the potential energy surface, which is not directly taken into account in the target function of machine learning. Note that such a large error in the elastic constants is also found in the analytic potential models (For example, Erhart and Albe compared the elastic constants of SiC, Si, and C obtained by various analytic potential models [9]). The accuracy of the elastic constants may be improved by including physical quantities related to the derivative of the potential energy in the reference data, e.g., as is adopted in the force-matching method [53]. Note that the ANN potential still has an advantage, even if the conventional potential functions can reproduce the structural and mechanical properties with a comparable accuracy. This is because ANN potentials are much more flexible and extendable than the flamework of the conventional potential models. For example, one can easily extend ANN potentials for Si-C systems to multi-species systems (e.g., Si-C-Al-B) without any special care or consideration, while conventional models are expected to require a substantial effort to determine a suitable function form for such multi-species systems.

### 3.3. Phase Stability of Crystalline SiC

**Figure 4:**
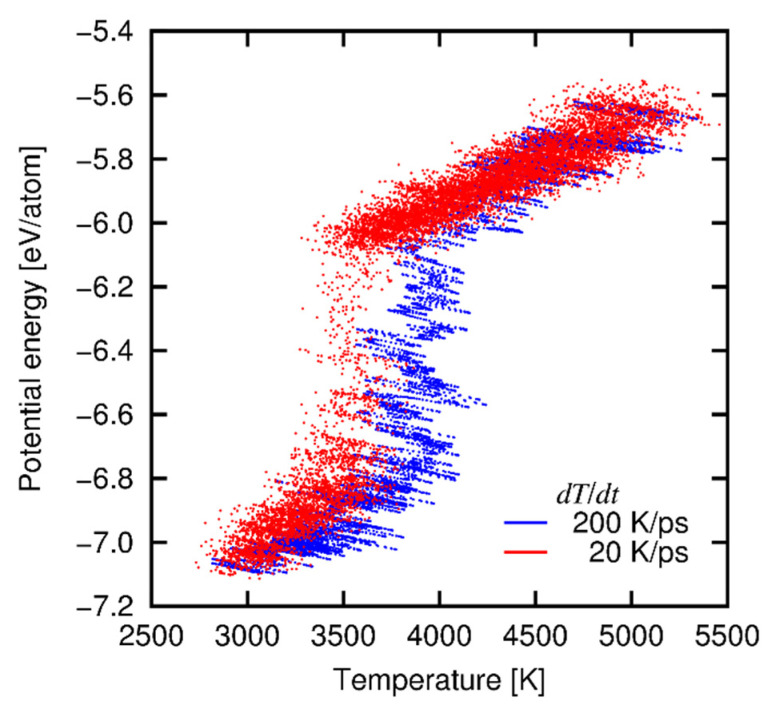
Relationship between potential energy and temperature at heating rates of 20 and 200 K/ps.

We examined the phase stability of crystalline SiC by conducting a melting test. A crystalline SiC model with 3C structure consisting of 512 atoms was prepared and heated at various heating rate *dT*/*dt* from 20 K/ps to 200 K/ps, where the cell size was adjusted in order that the thermal stress on the simulation cell was relaxed. Figure 4 shows the relationship between temperature and the potential energy per atom at 20 and 200 K/ps. It is found that the potential energy exhibits a discontinuous increase at certain temperature range (*T* ≈ 3500–4000 K), which is interpreted as the melting point *T*_m_. Note that *T*_m_ is dependent on the heating rate, i.e., *T*_m_ = *T*_m_(*dT*/*dt*), because of high heating rates in the MD simulation. Therefore, the *real T*_m_ should be evaluated by extrapolating as *dT*/*dt* → 0. By approximating the *T*_m_-*dT*/*dt* relationship with a quadratic function (See Appendix B), we estimated *T*_m_ → ≈3500 K. This value is in a fairly good agreement with the experimental decomposition temperature (≈3000 K) [54].

### 3.4. Structural Property of Amorphous SiC

**Figure 5:**
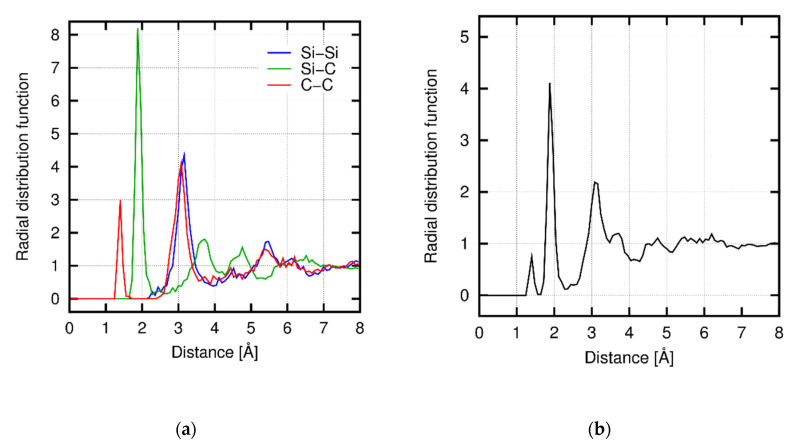
Radial distribution functions (RDFs) of amorphous SiC where (**a**) each contribution is shown separately, and (**b**) all combinations are included. All the RDFs are normalized by the global number density of atoms (regarding the corresponding pair type) and thus converge to 1 with the distance *r* → ∞.

To confirm the applicability of the ANN potential to the analysis of amorphous SiC, we prepared for an amorphous SiC structure model by the MD simulation of the melt-quench process. A 3C-SiC model with 512 atoms was fused at 4500 K and cooled to 300 K at the cooling rate *dT*/*dt* = 4.2 K/ps. Figure 5 shows the radial distribution function (RDF) of the amorphous SiC structure model at 300 K obtained by the melt-quench simulation. The profile of RDF is in a qualitatively good agreement with experiments [55,56], and typical peak profiles are well reproduced. Note that we observed partial recrystallization during the cooling process, and thus the RDF is dependent on the condition of the heating and cooling processes (e.g., an annealing process promotes recrystallization [55]).

## 4. Temperature-Dependence of Creep Properties of Amorphous SiC

### 4.1. Preparation of Simulation Cells

**Figure 6:**
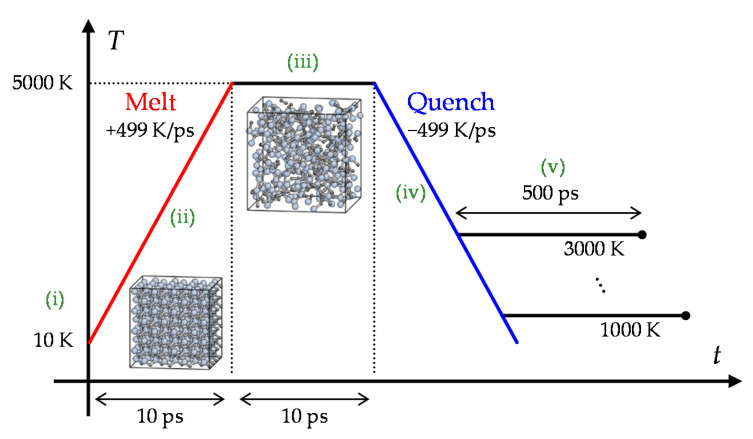
Procedure of preparation for amorphous structures.

**Figure 7:**
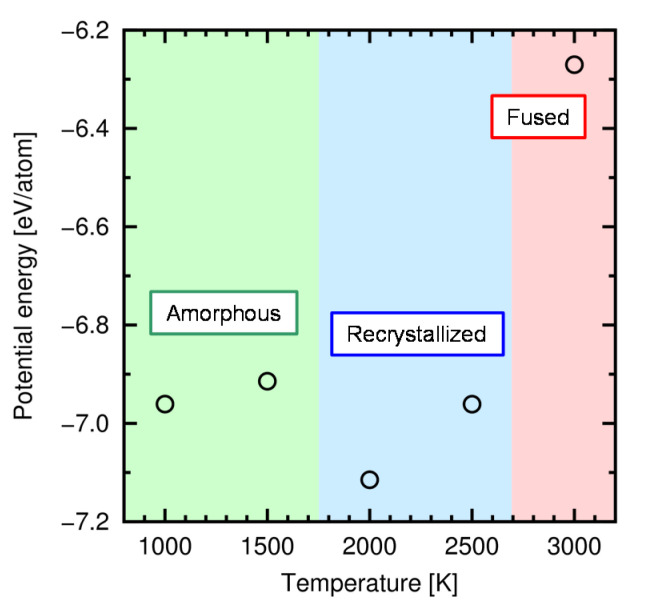
Potential energy of the quenched and relaxed structure at various temperatures (relaxation for 500 ps). Three regions indicate the resultant atomic structure at each temperature.

The amorphous structures for the creep tests were prepared for each target temperature. We set the target temperatures of *T* = 1000–3000 K, but the creep tests were actually examined only at *T* = 1000 K and 1500 K for the reason explained at the end of this subsection. While the real amorphization process is expected to be involved with the oxygen atoms, here we created the amorphous structures by the melt-and-quench method for simplicity. The structures were made in the following procedure (schematically shown in Figure 6): (i) A 3C-SiC supercell was prepared with 4 unit cells in each direction (512 atoms in total); (ii) The simulation cell was heated from 10 K to 5000 K (>*T*_m_) at a constant heating rate, *dT*/*dt* = 499 K/ps; (iii) The atomic structure was fully shuffled at 5000 K for 10 ps; (iv) The cell was quenched at a constant cooling rate, *dT*/*dt* = 499 K/ps, to the target temperature; (v) The cells were relaxed for 500 ps at the target temperature. During this process, isotropic stress was controlled to zero by adjusting the cell size. Note that the obtained structures are not completely amorphous because the structure is recrystallized during the quenching and relaxation processes to some extent. Especially, most part of the simulation cell was recrystallized during the relaxation process at 2000 K and 2500 K. Figure 7 shows the relationship between the potential energy of the relaxed structures and the temperature. From these results, we set the temperature range for the creep tests to *T* = 1000–1500 K, where the amorphous phase is likely to be kept.

### 4.2. Deformation Condition

We evaluate the creep properties of amorphous SiC, especially, the relationship between the stress and the strain rate. We considered two types of deformation conditions, i.e., constant-strain-rate and constant-stress conditions (hereafter *dε*/*dt*- and *σ*-constant conditions, respectively). While most experimental creep tests were conducted under a tensile loading condition, we applied compressive strain or stress to avoid void nucleation and brittle fracture. For the *dε*/*dt*-constant condition, true compressive strain was applied up to *ε* = 0.5 at constant true strain rates with the range of 2 × 10^−3^–5 × 10^−1^ ps^−1^. We conducted three sets of creep test along three different loading directions (i.e., *x*, *y*, and *z*) for each condition. For the *σ*-constant condition, we applied the compressive stress of 5–8 GPa at 1000 K and 4–7 GPa at 1500 K for 10 ps. As well as the case of the *dε*/*dt*-constant condition, we conducted the simulations three times along different loading directions for each condition. For both conditions, the normal stress components perpendicular to the loading direction were fully relaxed.

### 4.3. Results and Discussion

**Figure 8:**
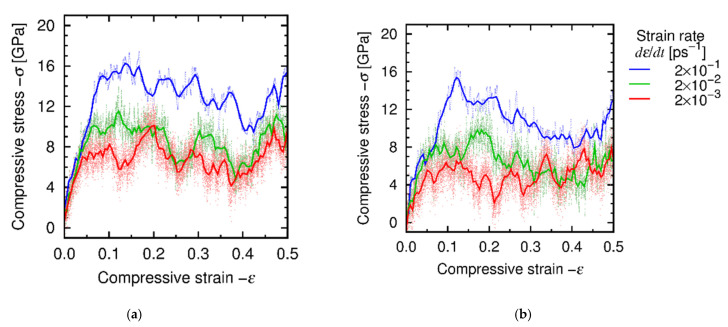
True stress-strain relationships under the *dε*/*dt*-constant conditions at (**a**) *T* = 1000 K and (**b**) *T* = 1500 K. The solid curves indicate the Bezier approximation.

**Figure 9:**
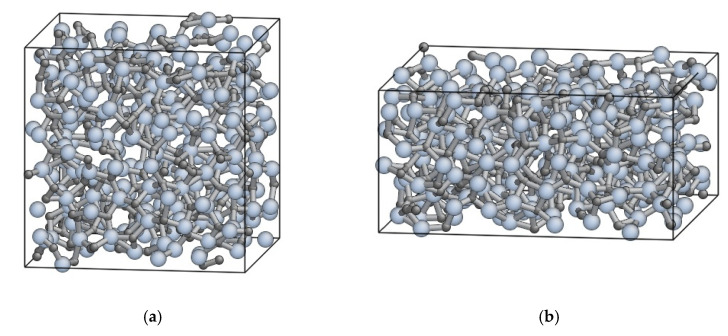
Atomic structure obtained at deformation test under the *dε*/*dt*-constant condition at *T* = 1500 K and *dε*/*dt* = 2 × 10^−3^ ps^−1^; (**a**) *ε* = 0.0 (before deformation), (**b**) *ε* = 0.5 (after deformation).

**Figure 10:**
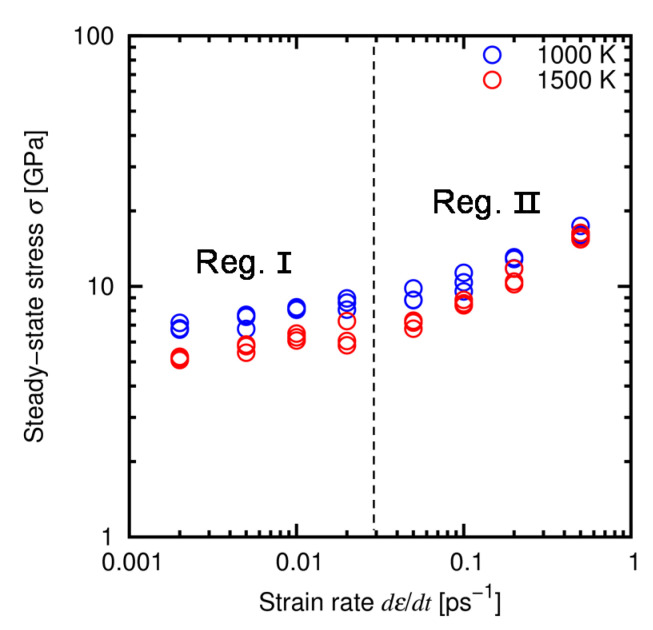
Relationship between stress and strain rate at the steady-state creep. The scales are shown in a logarithmic form. The vertical dashed line indicates the border of Regions I and II.

Figure 8 show the stress-strain relationship obtained under the *dε*/*dt*-constant conditions at *T* = 1000 K and 1500 K. For all the cases, the stress-strain relationship consists of two regions, i.e., the nearly elastic region at small strain and the steady-state region at large strain, and yielding occurs between those regions at *ε* ≈ 0.1. Relatively large fluctuation of stress in the steady-state region is attributed to the limitation of the simulation cell size. Figure 9 shows the atomic structure before and after deformation, obtained at *T* = 1500 K and *dε*/*dt* = 2 × 10^−3^ ps^−1^. This result indicates that the atomic structure is kept amorphous during the deformation test, which will be also confirmed by the RDFs later in this section. We evaluated the steady-state stress as the average value of stress over this region *ε* = 0.2–0.5. Figure 10 shows the relationship between stress and strain rate at the steady state. It is found that there are two regions at the low and high strain rates. The trend in both the regions follows the power law, which is qualitatively consistent with the experiments and thus supports the validity of the developed ANN potential. Hereafter we refer to the regions of low strain rates (*dε*/*dt* = 2 × 10^−3^–2 × 10^−2^ ps^−1^) and high strain rates (*dε*/*dt* = 5 × 10^−2^–5 × 10^−1^ ps^−1^) as Regions I and II, respectively.

**Figure 11:**
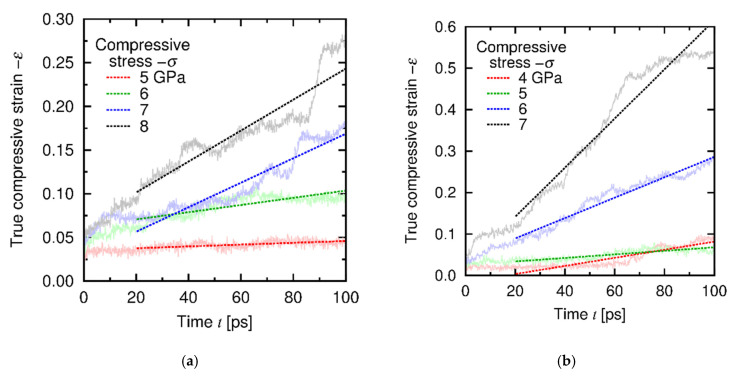
Temporal development of true compressive strain under the *σ*-constant conditions at (**a**) 1000 K and (**b**) 1500 K. The dashed lines indicate the linear approximations, and its slope is equal to the strain rate *dε*/*dt* at the steady state.

**Figure 12:**
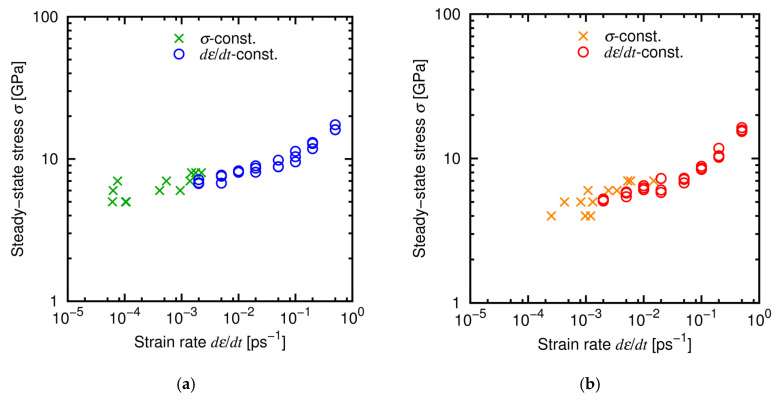
Relationship between the steady-state stress and the strain rate obtained under the *σ*- and *dε*/*dt*-constant conditions at (**a**) 1000 K and (**b**) 1500 K.

Figure 11 shows typical temporal developments of strain under the *σ*-constant conditions. Creep deformation was observed in all the cases, and we calculated the creep strain rates *dε*/*dt* from the *ε*-*t* relationship for 20–100 ps for each stress and temperature. Figure 12 shows the relationship between the stress and the strain rate at the steady state (the data of *dε*/*dt*-constant conditions are also shown for comparison and clarity). The results of the *σ*-constant condition are basically consistent with the *dε*/*dt*-constant results and found to belong to Region I. This result also shows that the *σ-dε*/*dt* relationship satisfies the power law in a wide range of *dε*/*dt*.

**Figure 13:**
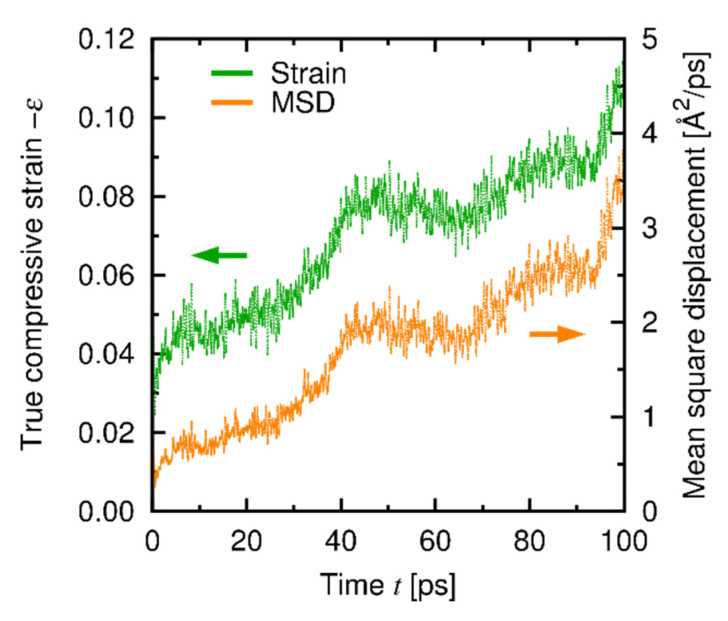
Temporal development of MSD and creep strain at Region I under the *σ*-constant condition (*σ* = 7 GPa, *T* = 1000 K). The MSD includes the contributions of both Si and C.

**Figure 14:**
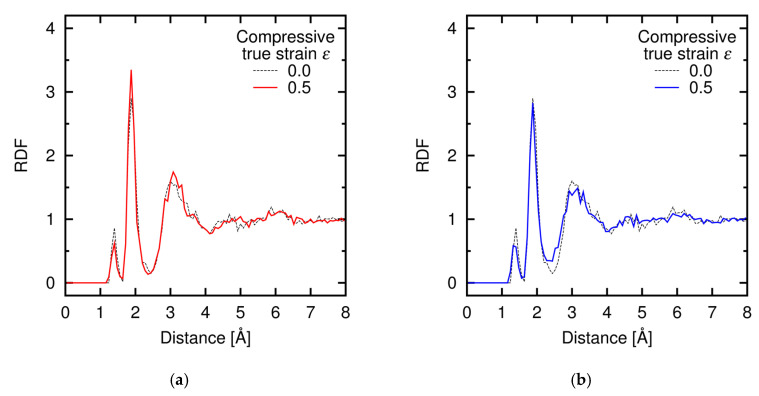
RDFs under *dε*/*dt*-constant conditions for all contributions (Si-Si, Si-C, and C-C) at (**a**) *dε*/*dt* = 2 × 10^−3^ ps^−1^ (Region I) and (**b**) *dε*/*dt* = 2 × 10^−1^ ps^−1^ (Region II) before and after deformation.

**Figure 15:**
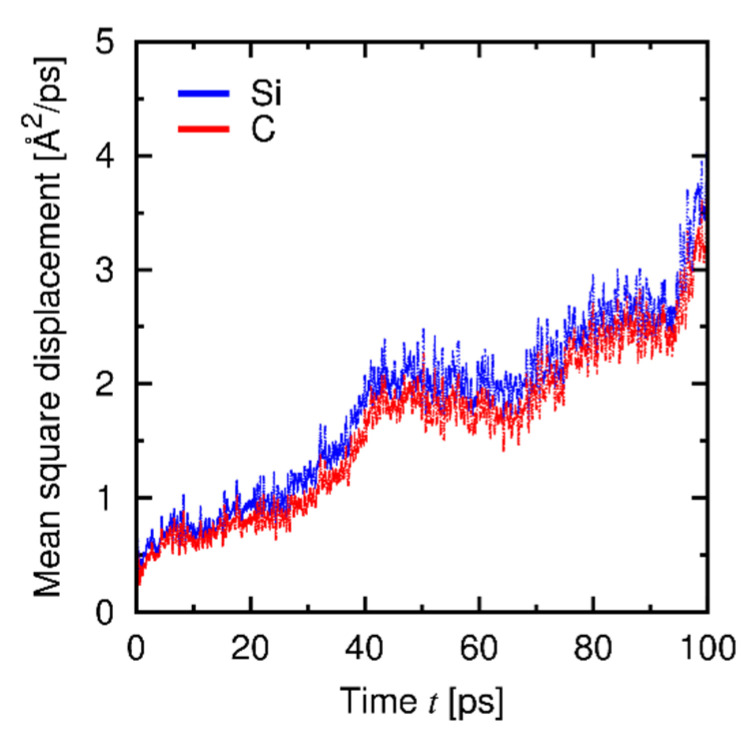
Temporal development of the individual MSDs for Si and C (*σ* = 7 GPa, *T* = 1000 K).

The creep behaviors at Regions I and II are expected to be based on different atomistic mechanisms. To reveal those mechanisms, we characterized the trend of change in the atomistic structures under deformation through the mean-square displacement (MSD) and the radial distribution function (RDF), which are related to the diffusion of atoms and local structural change, respectively. Figure 13 shows the temporal development of MSD and creep strain at Region I under the *σ*-constant condition, and Figure 14 shows the RDFs obtained under the *dε*/*dt*-constant conditions at Regions I and II before and after deformation. A clear correspondence between the trends in the MSD and the creep strain is found (Figure 13), while the RDFs at Region I remain nearly unchanged after deformation (Figure 14a). Therefore, the creep mechanism at Region I is attributed to diffusion of atoms in the amorphous structure. Figure 15 shows the temporal development of the individual MSDs for Si and C (decomposed from the MSD in Figure 13), which indicates that both the Si and C atoms are equally involved with the creep process. At Region II, in contrast, the profiles of the RDFs are partly affected by deformation (Figure 14b); especially, the peak at *r* = 3 Å of the Si-Si pair is significantly broadened after deformation (See Appendix C in detail). This result reveals that at Region II the deformation is faster than (or comparable to) relaxation of the local structure, and thus the creep behavior at Region II is characterized by the relaxation process in the local atomic structure. This type of deformation only occurs at a very high strain rate and thus is presumably negligible under a realistic condition. Note that the atomic structures are kept amorphous at both Regions I and II through the examination, as indicated by RDF profiles before and after deformation (Figure 14).

According to the experiments, the relationship between the stress and the strain rate at the steady state is described by a nonlinear viscoelastic model as follows [6,36]:

(9) $$\frac{d\varepsilon }{dt}=A\sigma^{n}\exp\left(\frac{-Q}{RT}\right),$$

where *A* is a material parameter. The parameters *n* and *Q* are the stress exponent and the activation energy, respectively. The constant *R* is the gas constant (*R* = 8.314 (J/mol)/K). The parameters *n* and *Q* are to be obtained via the present simulations. The *σ*-*dε*/*dt*-*T* relationship was fitted with this equation for each region from the data of the *dε*/*dt*-condition (Note that the data of the *σ*-condition were not used for approximation because of a relatively large deviation). The resultant fitting parameters are listed in Table 3 for each region, compared with the experimental results [5,6]. The stress exponents *n* are relatively large (especially at Region I) compared with the experimental values (typically *n* = 1–6 [36]) but within a reasonable range. The evaluated activation energies *Q* are significantly small compared with an experimental evaluation for a SiC fiber (Hi-Nicalon) [6] and the known elementary processes in SiC such as self-diffusion and grain-boundary diffusion [5]. The activation energy of creep in the SiC fiber is an apparent (nominal) value affected by various type of factors and elementary processes, and the diffusion in the amorphous phase can play an important role in those processes owing to its low activation energy. Note that the creep mechanism in the SiC fiber cannot be explained only via the known elementary processes mentioned above (i.e., self-diffusion and grain-boundary diffusion) because the activation energies of those processes are considerably higher than that of the SiC fiber, while those processes are presumably major factors. Thus, it is implied that the existence of a creep mechanism with a quite low activation energy, and the diffusion process in the amorphous phase is a possible cause of such low activation energy. It is a likely scenario that stress concentration at amorphous regions near the grain boundary results in a considerable creep deformation, owing to a quite large stress exponent *n*.

## 5. Conclusions

We developed an interatomic potential function based on the ANN model for the purpose of evaluating the high-temperature mechanical properties of Si-C system. The internal parameters in the model were optimized in order to reproduce the first principles results. It was confirmed by a series of verification simulations that the developed potential function can reproduce basic material properties such as the lattice constants and the stability of typical atomic structures. The elastic properties have a relatively large discrepancy, which should be improved in the future work by adopting advanced fitting approaches such as the force-matching method. The potential function was applied to the creep test of amorphous SiC under various loading conditions. As a result, it was found that there exist two regimes with different behaviors according to the strain rate. The mechanisms of creep deformation at the slow and fast deformation regions were attributed to atomic diffusion in the amorphous phase and relaxation of the local atomic structure, respectively. The activation energy of creep obtained from the present simulation is significantly lower than those of self-diffusion and grain-boundary diffusion. This result indicates the importance of diffusion in the amorphous phase.

While we dealt with a pure Si-C binary system, practical SiC/SiC composites include other various atomic species. To take them into account, it is necessary to develop a potential function for the multi-species system, based on the present ANN potential. In addition, a SiC/SiC component requires the environmental barrier coating system, which prevents SiC from chemical reaction with high-temperature vapor. Thus, it is also needed to investigate the mechanical properties of the interface between the SiC/SiC component and the coating layer, because a stress concentration and/or a thermal stress can be exerted near such a dissimilar interface. The MD results (material properties and/or deformation mechanisms) can be used for the larger-scale simulations, such as the phase-field and finite element analyses. Those subjects are left as future works.

## Figures and Tables

**Table 1:** Architectural parameters of basis function set. X in the combination type indicates the atomic species of centering atom (X = Si, C).

	2-Body	3-Body
Combination type	X-Si, X-C	Si-X-Si, Si-X-C, C-X-C
Number of terms per combination	16	4
Cutoff radius *r*_c_ [Å]	8.0	6.5

**Table 2:** Lattice constants (*a*, *c*), potential energy (*E*), and elastic constants (*C_ij_*) at equilibrium state of typical structures of SiC, Si, and C. Parameter *d* for C-graphene indicates the bond length. The column of “Exp.” and “MM” indicates the values obtained by experiments [47,48,49,50,51] and molecular mechanics simulation with conventional potential models (Tersoff (T94) [8], Erhart and Albe (EA) [9]), respectively.

			ANN	DFT	Exp.	MM T94 ^a^/EA
SiC	3C (zincblende)	*a* [Å]	4.371	4.379	4.3596 ^b^	4.280/4.359
		*E* [eV/atom]	−7.540	−7.532	-	−6.434/−6.340
		*C*_11_ [GPa]	425	384	390 ^b^	447/382
		*C*_12_ [GPa]	189	127	142 ^b^	138/145
		*C*_44_ [GPa]	190	233	256 ^b^	293/240
	2H (wurtzite)	*a* [Å]	3.082	3.091	3.076 ^c^	-
		*c* [Å]	5.103	5.073	5.048 ^c^	-
		*E* [eV/atom]	−7.539	−7.530	-	-
		*C*_11_ [GPa]	593	498	-	-
		*C*_33_ [GPa]	592	537	-	-
		*C*_12_ [GPa]	226	98	-	-
		*C*_13_ [GPa]	94	49	-	-
		*C*_44_ [GPa]	183	153	-	-
	4H	*a* [Å]	3.085	-	3.080 ^d^	-
		*c* [Å]	10.196	-	10.081 ^d^	-
	6H	*a* [Å]	3.088	-	3.080 ^d^	-
		*c* [Å]	15.294	-	15.098 ^d^	-
Si	diamond	*a* [Å]	5.486	5.469	5.429 ^e^	5.432/5.429
		*E* [eV/atom]	−5.418	−5.424	-	−4.63/−4.63
		*C*_11_ [GPa]	137	154	168 ^e^	143/167
		*C*_12_ [GPa]	66	57	65 ^e^	75/65
		*C*_44_ [GPa]	135	74	80 ^e^	119/60
C	diamond	*a* [Å]	3.572	3.572	3.567 ^f^	3.556/3.566
		*E* [eV/atom]	−9.127	−9.096	-	−7.473/−7.373
		*C*_11_ [GPa]	1336	1052	1081 ^f^	1010/1082
		*C*_12_ [GPa]	663	126	125 ^f^	169/127
		*C*_44_ [GPa]	785	551	579 ^f^	545/635
	graphene	*d* [Å]	1.436	1.424	1.42 ^f^	1.555/1.475
		*E* [eV/atom]	−9.261	−9.230	-	−5.314/−7.374

^a^ Calculated by Halicioglu [52]. ^b^ Lambrecht et al. [47]. ^c^ Merz and Adamsky [48]. ^d^ Lundqvist [49]. ^e^ Ref. [50]: Properties-Of-Silicon. ^f^ Ref. [51]: Numerical-Data.

**Table 3:** The fitted parameters for the relationship of the stress and the strain rate.

		*A* [GPa*^n^*∙ps^−1^] ^a^	*n*	*Q* [kJ/mol]
This work	Reg. I	7.7 × 10^−^^7^	7.5	50
-	Reg. II	2.7 × 10^−^^4^	3.2	13
Exp. [6]	Hi-Nicalon	-	2–3	193–423
Exp. [5]	self-diffusion (Si)	-	-	912
-	GB-diffusion (Si/C)	-	-	564/841

^a^ The unit depends on the parameter *n*.

**Figure A1:**
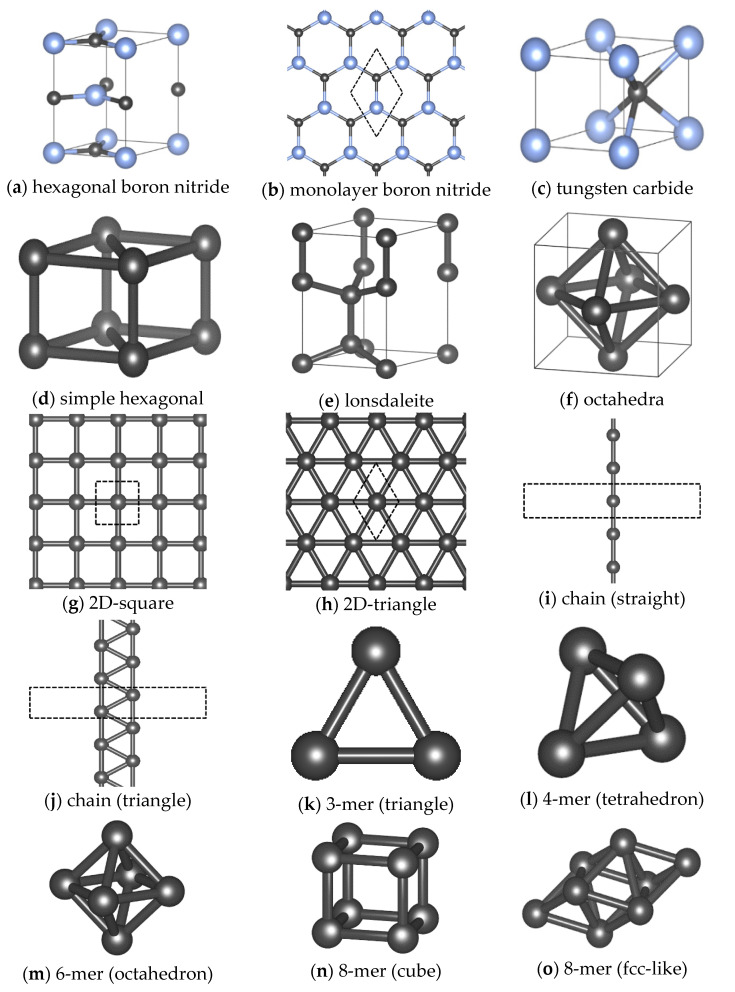
Atomic structures (untypical) used as reference structure. Thin solid and dashed lines indicate the unit cells.

**Figure A2:**
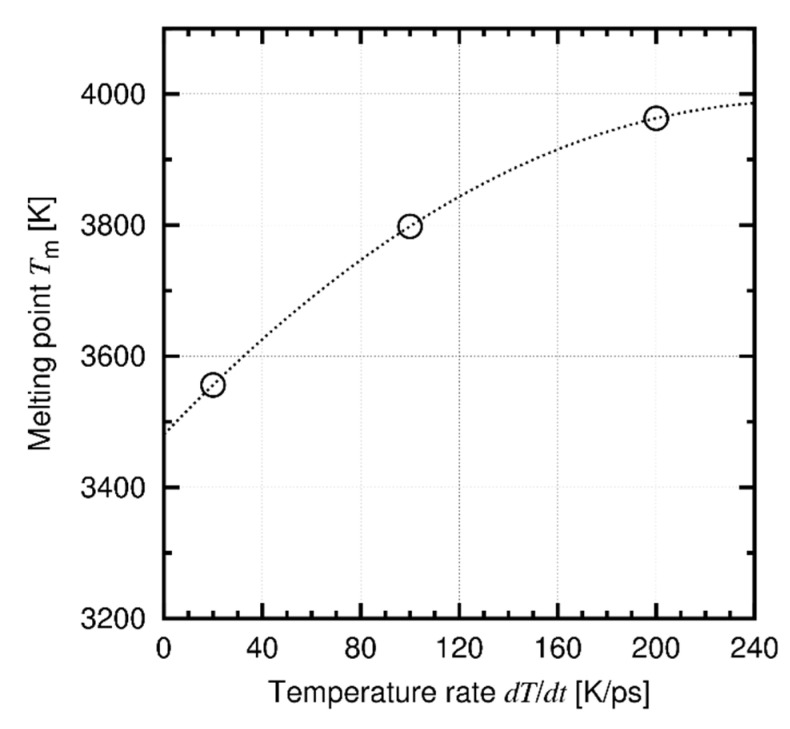
Melting temperature as a function of heating rate. The dashed curve indicates a quadratic approximation.

**Figure A3:**
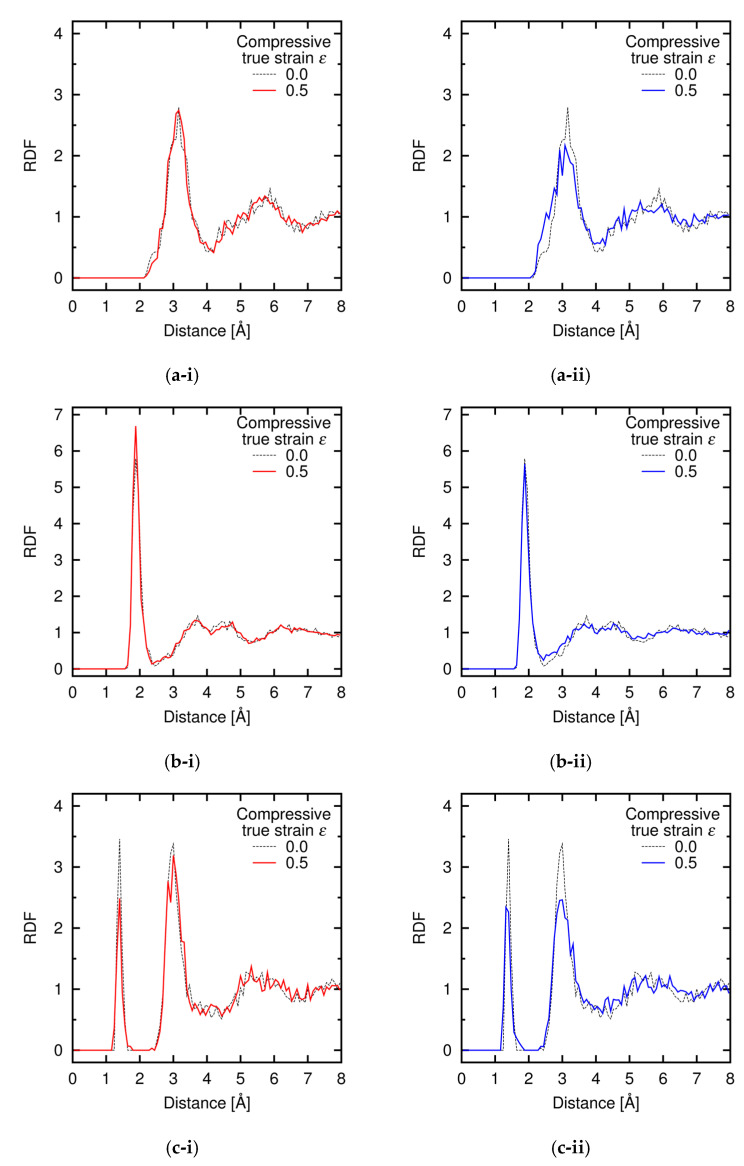
RDFs under *dε*/*dt*-constant conditions for (**a**) Si-Si, (**b**) Si-C, and (**c**) C-C at (**i**) *dε*/*dt* = 2 × 10^−3^ ps^−1^ (Region I) and (**ii**) *dε*/*dt* = 2 × 10^−1^ ps^−1^ (Region II) before and after deformation.

## Data Availability

Data (the developed ANN potential) available on request.

## Appendix A. Typical Reference Crystal Structures

The typical reference crystal structures are listed in Table A1 (several non-crystal molecules are also included, e.g., dimer, trimer, etc.). The *x*, *y*, and *z* axes in “Deformation Mode” indicate the [100], [010], and [001] directions for the cubic structures, and the $\left[2\bar{1}\bar{1}0\right]$, $\left[01\bar{1}0\right]$ and [0001] directions for the hexagonal structures, respectively. The deformation mode “*XY*” indicates that the *X* component is deformed in the *Y* direction (thus, the case of *X* = *Y* means a normal strain, and otherwise shear strain). The combination of *XX* and *YY* indicates the coupling of two deformation modes. The mode “iso” is the isotropic deformation, i.e., *xx* + *yy* + *zz*. The deformation modes with the notation of “*d* = *A*–*B* Å” indicate that the interatomic distance from the first neighbors was varied instead of changing the strain (mainly applied to non-crystal systems). The atomic structures that are not typical or that we defined are visualized in Figure A1.

**Table A1:** Typical crystal structures and deformation modes considered as reference data. The structures with an asterisk (“*”) are visualized in Figure A1.

System	Structure	Deformation Mode
SiC	zincblende (3C)	iso, *xx*, *xx + yy*, *xx − yy*, *xy*
	wurtzite (2H)	iso, *xx*, *yy*, *zz*, *xx + yy*, *xx − yy*, *zx*, *zy*
	rock salt	iso, *xx*, *xx + yy*, *xx − yy*, *xy*
	cesium chloride	iso, *xx*, *xx + yy*, *xx − yy*, *xy*
	hexagonal boron nitride *	iso, *xx*, *yy*, *zz*, *xx + yy*, *xx − yy*, *zx*, *zy*
	monolayer boron nitride *	*d* = 0.80–3.00 Å
	fluorite (SiC_2_, CSi_2_)	iso, *xx*, *xx + yy*, *xx − yy*, *xy*
	tungsten carbide *	iso, *xx*, *yy*, *zz*, *xx + yy*, *xx − yy*, *zx*, *zy*
	dimer	*d* = 0.80–4.00 Å
Si	diamond	iso, *xx*, *xx + yy*, *xx − yy*, *xy*
	graphene	*d* = 0.80–3.00 Å
	bcc	iso, *xx*, *xx + yy*, *xx − yy*, *xy*
	fcc	iso, *xx*, *xx + yy*, *xx − yy*, *xy*
	sc	iso, *xx*, *xx + yy*, *xx − yy*, *xy*
	simple hexagonal *	iso, *xx*, *yy*, *zz*, *xx + yy*, *xx − yy*, *zx*, *zy*
	octahedra *	iso, *xx*, *xx + yy*, *xx − yy*, *xy*
	2D-square *	*d* = 0.90–3.00 Å
	2D-triangle *	*d* = 0.90–3.00 Å
	chain (straight) *	*d* = 0.90–4.00 Å
	dimer	*d* = 1.00–4.00 Å
C	graphene	*d* = 0.80–3.00 Å
	graphite (α, β)	iso, *xx*, *yy*, *zz*, *xx + yy*, *xx − yy*, *zx*, *zy*
	diamond	iso, *xx*, *xx + yy*, *xx − yy*, *xy*
	lonsdaleite *	iso, *xx*, *yy*, *zz*, *xx + yy*, *xx − yy*, *zx*, *zy*
	bcc	iso, *xx*, *xx + yy*, *xx − yy*, *xy*
	fcc	iso, *xx*, *xx + yy*, *xx − yy*, *xy*
	hcp	iso, *xx*, *yy*, *zz*, *xx + yy*, *xx − yy*, *zx*, *zy*
	sc	iso, *xx*, *xx + yy*, *xx − yy*, *xy*
	simple hexagonal *	iso, *xx*, *yy*, *zz*, *xx + yy*, *xx − yy*, *zx*, *zy*
	octahedra *	iso, *xx*, *xx + yy*, *xx − yy*, *xy*
	2D-square *	*d* = 0.80–3.00 Å
	2D-triangle *	*d* = 0.80–3.00 Å
	chain (straight) *	*d* = 0.80–4.00 Å
	chain (triangle) *	*d* = 0.80–3.00 Å
	dimer	*d* = 0.80–4.00 Å
	3-mer (triangle) *	*d* = 0.80–3.00 Å
	4-mer (tetrahedron) *	*d* = 0.80–3.00 Å
	6-mer (octahedron) *	*d* = 0.80–3.00 Å
	8-mer (cube) *	*d* = 0.80–3.00 Å
	8-mer (fcc-like) *	*d* = 0.80–3.00 Å

## Appendix B. Approximation of Melting Point

Figure A2 shows the melting point *T_m_* as a function of temperature rate *dT*/*dt* for 3C SiC. The data points were fitted with a quadratic function, i.e., *T*_m_ = *A*⋅(*dT*/*dt*)^2^ + *B*⋅(*dT*/*dt*) + *C*. Then the fitting parameter *C* is equal to *T*_m_ at *dT*/*dt* → 0.

## Appendix C. Radial Distribution Function during Deformation

Figure A3 shows the RDFs obtained under the *dε*/*dt*-constant conditions at Regions I and II before and after deformation for each atomic pair (decomposed from Figure 14). The distribution of the Si-Si pair is affected by deformation at the high strain rate (Reg. II; Figure A3(a-ii)), while all the distributions are nearly unchanged at the low strain rate (Reg. I; Figure A3(a-i)–(c-i)). This result implies relaxation of the local structure as a major contributor of creep deformation at Reg. II (see main text).
